# fMRI Artefact Rejection and Sleep Scoring Toolbox

**DOI:** 10.1155/2011/598206

**Published:** 2011-03-13

**Authors:** Yves Leclercq, Jessica Schrouff, Quentin Noirhomme, Pierre Maquet, Christophe Phillips

**Affiliations:** ^1^Cyclotron Research Centre, University of Liège, 4000 Liège, Belgium; ^2^Department of Neurology, University of Liège, 4000 Liège, Belgium; ^3^Department of Electrical Engineering and Computer Science, University of Liège, 4000 Liège, Belgium

## Abstract

We started writing the “fMRI artefact rejection and sleep scoring toolbox”, or “FA*𝕊*T”, to process our sleep EEG-fMRI data, that is, the simultaneous recording of electroencephalographic and functional magnetic resonance imaging data acquired while a subject is asleep. FA*𝕊*T tackles three crucial issues typical of this kind of data: (1) data manipulation (viewing, comparing, chunking, etc.) of long continuous M/EEG recordings, (2) rejection of the fMRI-induced artefact in the EEG signal, and (3) manual sleep-scoring of the M/EEG recording. Currently, the toolbox can efficiently deal with these issues via a GUI, SPM8 batching system or hand-written script. The tools developed are, of course, also useful for other EEG applications, for example, involving simultaneous EEG-fMRI acquisition, continuous EEG eye-balling, and manipulation. Even though the toolbox was originally devised for EEG data, it will also gracefully handle MEG data without any problem. “FA*𝕊*T” is developed in Matlab as an add-on toolbox for SPM8 and, therefore, internally uses its SPM8-meeg data format. “FA*𝕊*T” is available for free, under the GNU-GPL.

## 1. Introduction

“FA*𝕊*T” stands for “fMRI artefact rejection and sleep scoring toolbox”. We, researchers from the Cyclotron Research Centre, University of Liège, Belgium, started writing this set of tools to analyze our sleep EEG-fMRI data, that is, both electroencephalographic (EEG) and functional magnetic resonance imaging (fMRI) data acquired simultaneously while the subject is asleep. The joint acquisition of EEG and fMRI data allows the integration of electric and haemodynamic information about brain activity [1]. This is a requirement, for example, in neuroimaging sleep studies as sleep activity can only be derived from the EEG signal, while fMRI allows the localization of haemodynamic signal variation throughout the brain volume [2, 3]. Nevertheless, when processing such data, one has typically to tackle three crucial issues.

**Handling Large Data Sets**

Reviewing long multichannel continuous recording of M/EEG (magneto- and/or electroencephalographic) activity is cumbersome as it usually involves displaying and manipulating (exploring, comparing, chunking, appending, etc.) large data sets, up to several gigabytes (Gb) for hour long recordings.

**fMRI Artefact Rejection**

When recording EEG and fMRI data simultaneously, the EEG signal is contaminated by artefacts induced by the gradient switching and high static field of the MR scanner. The rejection of these artefacts is very challenging. If the EEG data are not averaged afterwards, that is, for continuous or single trial analysis, then any inaccuracies in this rejection may have a large and negative impact on the results.

**Scoring Data**

Scoring continuous M/EEG recordings, such as is common with sleep recordings, is a tedious task, as the scorer has to manually browse through the entire data set and give a “score” to each time-window displayed.

As far as we know, FA*𝕊*T is the only freely available software package that can efficiently deal with those three issues. Moreover, the tools provided can also be tailored for one's own need, and new features can easily be added (as additional Matlab functions), leading to a flexible toolbox for anyone dealing with (long) M/EEG recordings, EEG-fMRI data and/or (sleep) signal scoring.

We chose to implement our ideas as an add-on toolbox for SPM8 ([4] and Litvak et al. in this issue) and not, for example, an add-on for EEGlab ([5] and Delorme and Makeig in this issue), for two practical reasons, stemming from our original sleep EEG-fMRI data.

**A Single Processing Platform**

We found it more convenient to process both EEG and fMRI data within the same software suite. Since we were already using SPM to process our fMRI data, we decided to add EEG tools (continuous recording visualization/handling, fMRI artifact rejection and sleep scoring) to the SPM8 package.

**The Data Format**

Thanks to the memory mapping feature of the SPM8-meeg data format (see Section 2.1), whatever the size of the data set saved on the computer hard disk, only the bits required for the current operation are actually loaded in memory. This offers a quick and transparent access to data set up to several gigabytes, even on a standard computer (32 bits machine and less than 4 Gb of RAM).

The latter point is absolutely crucial for us, and only SPM8 provides this feature at the moment. FA*𝕊*T thus internally uses the open SPM8-meeg data format. The conversion from the original data format to that of SPM8 can be performed directly by FA*𝕊*T or through SPM8, see Section 2.1. Note that it is not necessary to master the whole SPM8 package to use FA*𝕊*T. Once in the appropriate format, M/EEG data can then be easily visualized and manipulated; see Section 3.1.

Specifically, for EEG-fMRI acquisitions, FA*𝕊*T can operate directly on the raw data acquired with a “brainamp MR” system (BrainProducts Gmbh, Gilching, Germany) and includes the well-known “averaged artefact substraction” (AAS) method [6] for the “gradient artefact” rejection, as well as the recently published “constrained independent component analysis” or cICA method [7] for the rejection of the “pulse artefact”, see Section 3.2. Other classic methods for the “pulse artefact” rejection are also available. Finally, an easy GUI is available for the manual scoring of continuous M/EEG data: sleep stages, for sleep recordings, or any other “stage”; for other types of data, see Section 3.3. Some statistics and sleep-specific features can also be automatically extracted.

 FA*𝕊*T can be operated in 2 ways: via user-friendly GUIs or the command line. The GUIs let the user select the data to process, and tune various parameters and options for each tool. The “default” parameter values can also be modified by editing a single “default” file, allowing user- or site-specific settings. The command line approach allows the scripting of operations, for example, to automatically process several recordings with one execution of a Matlab script.

 FA*𝕊*T is distributed for free, under the GNU-GPL, and available for download at the following address: http://www.montefiore.ulg.ac.be/~phillips/FASST.html. It comes without any warranty: you should use it at your own risk. A manual detailing FA*𝕊*T features and possibilities is also available here: http://www.montefiore.ulg.ac.be/~phillips/FASST_manual.pdf.

## 2. Software Characteristics

FA*𝕊*T is an add-on to the popular SPM8 software and is written in Matlab, with a few routines written in C/C++ but interfaced with Matlab. Since Matlab is a high level multiplatform computing language, only the few routines written in C/C++ need to be compiled for a specific operating system. So far, those routines were compiled for Windows XP only, but some operating systems (like Windows 7 and Mac OS X) will be directly supported by distributing the compiled routines. For the other OSs (like Unix) a simple compilation script will be made available in the next release. In order to work properly, FA*𝕊*T, therefore, needs to have the following 2 softwares installed:

- a recent version of Matlab. We used version 7.5 (R2007b) (any later Matlab version should *in theory* work too) to develop FA*𝕊*T, as well as Matlab “signal processing toolbox” (for some filtering functions though this requirement will be lifted in the next release),
- the latest SPM8 version. FA*𝕊*T relies on the SPM8-meeg data format (see Sections 2.1 and 2.2) and also uses some M/EEG-specific routines.

Note also that FA*𝕊*T, on top of relying on Matlab and SPM8, includes a few routines from the following three other freely available Matlab toolboxes:

- EEGLAB ([5] and Delorme and Makeig in this issue), mainly for a few functions used by the constrained ICA “pulse artefact” rejection, available from http://sccn.ucsd.edu/eeglab/,
- the FMRIB plugin for EEGLAB [8], for the electrocardiographic (ECG) peak detection and classic “principal component analysis” (PCA, which is sometimes refered as the “optimal basis set” (OBS) approach) and “Gaussian mean” (a variation of the AAS method) pulse artefact rejection methods, available from http://www.fmrib.ox.ac.uk/eeglab/fmribplugin/index.html,
- the “mutual information computation” package [9], for the selection of correction matrices during cICA “pulse artefact” rejection, available from http://penglab.janelia.org/proj/mRMR/,

These additional routines are already included in FA*𝕊*T and do not need to be downloaded separately. We, therefore, thank their authors for letting us use and distribute their work.

### 2.1. Data Format

FA*𝕊*T internally works with SPM8-meeg data format which stores M/EEG data in two separate files: a  .mat header file and a  .dat binary file. contains all the information about the data (channel names and types, sampling rate, stimuli, etc.), and the binary file stores the data themselves as a raw list of numbers. For a thorough description of SPM8-meeg data format, one should have a look at SPM8 documentation, but it is worth mentioning here the key feature of SPM8-meeg data format used by FA*𝕊*T: the whole data set is not loaded into memory, but only the header content. Then only the “window”, over time and/or channels, of data required for the current operation (e.g., displaying the signal from 10 channels over 20 seconds) is loaded. The trick is that the data file on disk is memory mapped into Matlab, such that it can be accessed transparently, as a regular variable, without eating up all the memory. For example, an EEG-fMRI sleep recording of 4 hours weights about 9,5 Gb of EEG data (72 channels × 4 hours × 3600 seconds/hour × 5000 Hz sampling rate × 2 bytes/sample) but can be displayed and manipulated without any problem. Any new data file generated by FA*𝕊*T is of course stored in the same format. Additional information generated and used by FA*𝕊*T, such as the sleep score encoded by one or more users, are simply added to the header data structure and do not interfere with SPM8 machinery.

Data conversion or importation is always an issue in EEG and MEG, since each company enjoys his own specific (and usually proprietary) format. With FA*𝕊*T, there are 2 ways to import the data in the right format, either with the SPM8 “convert” function or directly in FA*𝕊*T.

#### 2.1.1. SPM8 Data Conversion

SPM8 relies on the “fieldtrip” (FT) toolbox [10] and Oostenveld et al. in this issue to read in and convert pretty much any existing EEG/MEG data formats. SPM8-FT generally reads in the original header information/file(s) and creates the  .mat header file, then goes through the data file(s) and creates the associated binary file. This approach is very robust and probably the safest.

Nevertheless, SPM8-FT conversion may take some time for large data sets, as data are read in and then written on disk in the new  .dat file. Moreover, depending on the way raw original data are stored, SPM8-FT can more than double the size of your original data on disk: for example, raw EEG data in INT16 format, that is, 2 bytes per sample, are written in FLOAT, that is, 4 bytes per sample, and will thus double the disk space usage.

#### 2.1.2. FA*𝕊*T Data Conversion

 FA*𝕊*T can use its own specific importation routines, but this approach is much less exhaustive than SPM8-FT in terms of supported data formats. So far only Brain Products (Brain Products GmbH, Gilching, Germany) is directly supported (Raw-EGI (Electrical Geodesics Inc., Eugene, OR, USA) and  .edf data formats import are only in beta version.) Yet, FA*𝕊*T specific approach has 2 advantages. First, these data can be directly selected via the FA*𝕊*T GUI, and they will be converted “on the fly”, that is, no need to launch SPM8. Second, brain products EEG data are imported without generating a second data file. Practically, the  .vhdr and  .vmrk header files are directly read in by FA*𝕊*T and translated into SPM8-meeg .mat file, which is then directly linked to the original binary data file, without reading-converting-writing the data themselves. Moreover, FA*𝕊*T will also (try to) recover and save the “real-world” beginning time of the recording, that is, the computer clock time of the recording, from the original data. This is useful when comparing, appending and chunking files (see Sections 3.1.2 and 3.1.3).

There is, thus, no “import” button in FA*𝕊*T and EEG data acquired with a Brain Products amplifier can be directly selected in the GUI: the (header) conversion will take place automatically. This is very fast, as only “administrative” bits of information about the data (including the triggers) are converted, and disk space efficient, as the data binary file is not copied. These two features are particularly useful for simultaneous EEG-fMRI recordings where data files easily reach several Gbs. Other data formats should first be converted using SPM8-FT functionalities.

### 2.2. Channel Definition

Channel definition (name, type, and 2D-location) is in line with that of SPM8, which also offers GUI facilities to easily edit channel and data information. The goal of FA*𝕊*T's “channel definition” is not to import subject specific information, such as importing channel location file (this actually can be done within SPM8), but rather to add some features to the standard SPM8 format: this allows mainly the on-the-fly display of simple bipolar montage (like horizontal or vertical EOG's) alongside M/EEG channels and the use of different channel scalings, for example, for M/EEG and ECG/EMG/EOG channels. Most common channel names are already available within the toolbox, but any laboratory or experiment specific setup can be added: The default electrode/sensor setup are defined in a “electrode defaults” file, which is a simple Matlab script easy to edit.

## 3. Main Functions of the Toolbox

The following tools and features are available in the current version of FA*𝕊*T:

- handling tools: displaying one M/EEG data file, comparing multiple M/EEG data files channel by channel, appending M/EEG data files, chunking a time window from an M/EEG data file, and computing and displaying the spectrogram of one M/EEG data file,
- EEG-fMRI artefact rejection tools: gradient artefact rejection (AAS method) and pulse artefact rejection (AAS, “Gaussian mean” and cICA methods),
- sleep specific tools: manual sleep scoring, spectral power calculations, slow wave detection and propagation, and sleep statistics.

### 3.1. General Tools

As mentioned previously, these are tools to display, review, compare, process, manipulate, and handle (long) continuous data files, containing EEG, MEG, EOG, ECG, EMG, or any other sampled signal.

#### 3.1.1. Displaying One M/EEG Data File

**Figure 1:**
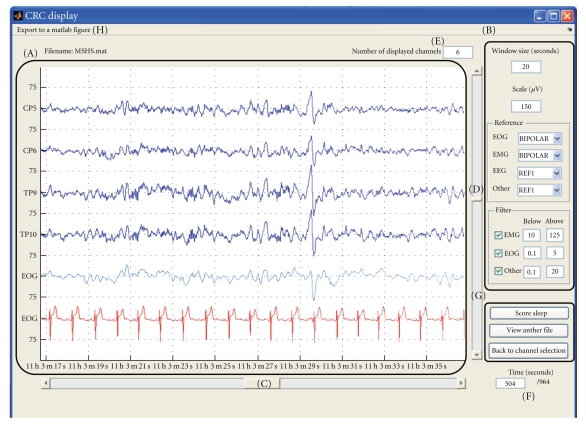
Main signal display window: (A) main display, (B) display options (see text for details), (C) time scrolling bar, (D) channel scrolling bar, (E) number of channels to display at once, (F) time in seconds at the beginning of display, (G) change channels or file to display, or start “sleep scoring”, and (H) exportation of the current main display to a new Matlab figure.

One continuous M/EEG recording (of any length) can be easily displayed and rapidly browsed through; see Figure 1. All or any subset of channels can be selected, then the channel signals are displayed in the main central box. There are two scrolling bars: one to quickly browse throughout the data over time and another one to browse through subsets of selected channels.

To help visualization, standard unipolar channels are displayed in blue, bipolar channels in green, and automatically rescaled channels are shown in red. The list of channels that are automatically rescaled and the bipolar montages are specified in the “electrode defaults” file. For example, electrocardiographic (ECG) signal has a much larger amplitude than EEG, and thus ECG channels should be scaled differently for a convenient display. Several other options are available through the GUI.

- The number of channels per screen, time window (in seconds) displayed, and channel scale (in *μ*V) can be modified at any time. The numbers on the side of the main display (75 in Figure 1) indicate the scale used for the EEG channels in *μ*V, except for the EOG/EMG/ECG channels which have a fixed scale defined in the default file.
- Reference can be modified through a pulldown menu. For the EEG channels, this reference may be any other channel, the mean of all the EEG channels or the mean of the two mastoids (called M1 and M2). For the EOG and EMG channels there is an additional “Bipolar” choice for the reference.
- A different bandpass filter can be applied to the different types (EMG, EOG or “Other”) of channels.
- The power spectrum of the displayed signal can be directly computed for one channel via a “right-click” pulldown menu in the main display over the specific channel. The resulting spectrum is then shown in a separate Matlab figure.

Note that the rereferencing and filtering are performed only “on-display” and the original data stored on disk are left untouched. There is therefore no need to perform these pre-processing steps before visualizing the data. In fact these features let the user explore the effect of filtering or rereferencing on the displayed data.

#### 3.1.2. Comparing Multiple M/EEG Data Files

**Figure 2:**
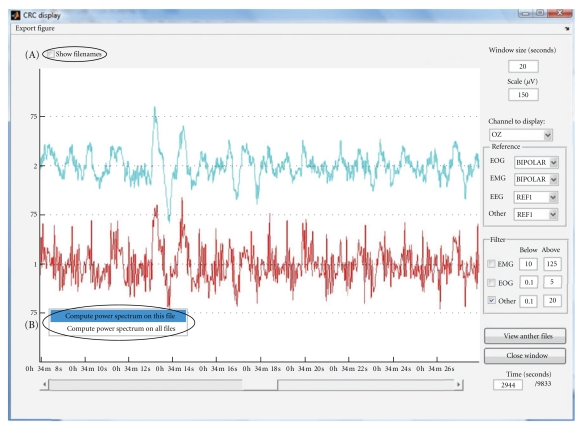
Multiple files comparison GUI: (A) toggle filename display and (B) compute power spectrum of one or all channels.

This tool is designed to display the same channel from multiple M/EEG files. It can obviously be used to compare the results of different artefact correction methods applied to the same data set or to visually check the effect of any processing applied to a continuous data set. For example, Figure 2 shows the same signal before (bottom) and after (top) pulse artefact correction. Only one channel can be displayed at one time and the displayed channel is selected via a pulldown menu.

The routine also checks the “real-world” beginning time, that is, the computer clock time of the recording, of each data set and aligns the different M/EEG time series in consequence. If the beginning time was not imported from the raw data; all files are assumed to begin at the same time with the first sample. The power spectrum of the displayed signals can also be directly estimated and shown in a separate window.

#### 3.1.3. Appending and Chunking M/EEG Data Files

The appending tool is designed to append two separate M/EEG files into a single one. This is particularly useful if recording was (accidentally) interrupted but the different data sets should be considered as one single “recording session”. If the “real-world” recording time is available, then the file order is automatically determined and any time gap between the end of the first file and the beginning of the second is filled with zeros. Otherwise the data will simply be appended one directly after the other.

“Chunking” is the opposite of “appending”, and lets the user cut out an episode out of a large M/EEG file to save it as a separate data file. This can be useful if one wants to study a specific episode of activity such as sleep stages and epileptic discharge. The beginning and end of the new file can be defined by markers (or triggers) or by time (relative to the beginning of the file or in “real-world” time).

#### 3.1.4. Computing and Displaying the Spectrogram of One M/EEG Data File

Using the Welch periodogram method, the spectrogram of one whole data set can be computed. The output is saved into a time-frequency data file, also in an SPM8-meeg compatible format. Before the computation itself, the data are bandpass filtered. Then, the spectrogram is computed over overlapping time windows. 

**Figure 3:**
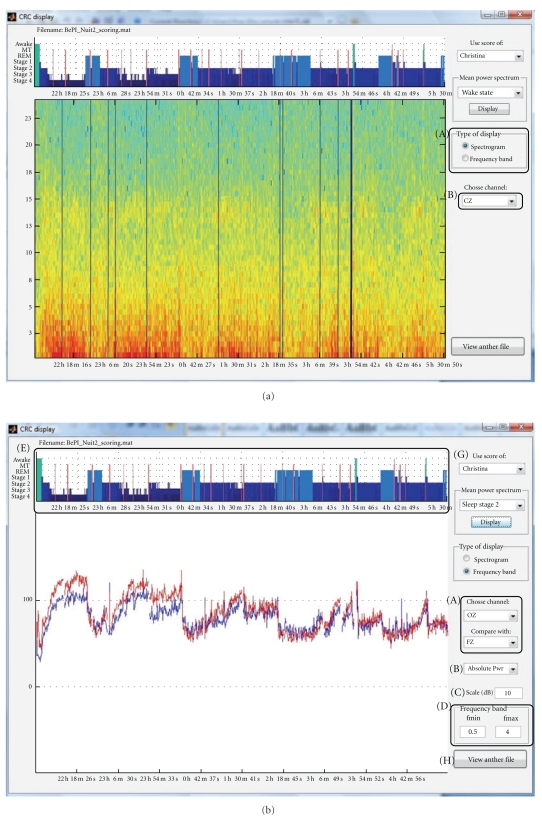
Main display of spectrogram GUI. (a) “Spectrogram display”: (A) display mode toggle and (B) channel selection and (b) “frequency band display”: (A) channel(s) selection, (B) scaling type, (C) scale for display, (D) frequency band, (E) night hypnogram, (F) mean power spectrum for a specific sleep stage, (G) selection of the hypnogram scorer, and (H) selection of another data file.

Once calculated, the spectrogram can be displayed in two ways: “spectrogram” is the time-frequency representation of one channel and a pulldown menu is used to select the channel to display (see Figure 3(a)). In the “frequency band” display mode, the evolution of the power in a specified frequency band is displayed for one or two channels (see Figure 3(b)).

### 3.2. EEG-fMRI Artefact Rejection Tools

 When EEG is recorded during fMRI acquisition, two types of artefacts are induced on top of the neural EEG signal.

- The “gradient artefact” (GA) is due to the gradient switching of the imaging sequence of the MR scanner [6].
- The “pulse artefact” (PA) is due to the interaction between the static field of the MR scanner and the heartbeats [11]. This artefact is present even if no fMRI data are acquired.

One should always suppress the gradient artefact before the pulse artefact, as the amplitude of the former is several orders of magnitude larger than the latter.

#### 3.2.1. Gradient Artefact Rejection

The GA is removed using the “average artefact subtraction” (AAS) method developed by Allen et al. 2000 [6]. AAS estimates the shape of the GA over a “repetition time” (TR, or the time elapsed between the acquisition of two fMRI volumes) by averaging the signal over several (30 by default) contiguous fMRI volume acquisitions. This “averaged artefact” is estimated for each TR and subtracted from the recorded EEG signal. The efficiency of the AAS approach relies on the stationarity of the GA picked in the EEG signal. This stationarity can be enforced by synchronizing the clocks of the EEG amplifier(s) with that of the MR scanner. This is a crucial point, and any user applying this algorithm to data acquired without clock synchronization may (and most certainly will) have improper GA rejection.

The beginning of each fMRI volume can be specified either by triggers sent from the scanner (the safest option), or by using the sequence TR and automatically detecting the scanning episode (less reliable). When triggers (one per fMRI volume or slice) are available, then the correction will be based exclusively on these. If they are not available, the user can manually specify the beginning and end (in seconds from the beginning of the of EEG file) of the EEG episode to correct. This interval can also be automatically detected using a simple amplitude criteria: a stretch of EEG data with the mean (over a specified time window) absolute signal amplitude of a specific channel above some threshold (by default, one second, the first channel and 350 *μ*V), then this is considered as an artefacted episode to be corrected. The TR provided is then used for the AAS correction. Finally, the sampling frequency of the original file (typically 5 kHz) being usually higher than necessary for further processing, the data are downsampled during the process (to 500 Hz by default).

#### 3.2.2. Pulse Artefact Rejection

The PA is induced by the interaction between the heartbeats of the subject, which induce small movements, and the static field of the MR scanner. The PA is more difficult to remove than the GA because of its nonstationarity: it varies from heartbeat to heartbeat! Moreover, its amplitude (a few 10s of *μ*V in our 3T scanner) and power spectrum (main frequency around 1-2 Hz and higher harmonics) render it difficult to disambiguate and filter out from genuine EEG signal.

**Figure 4:**
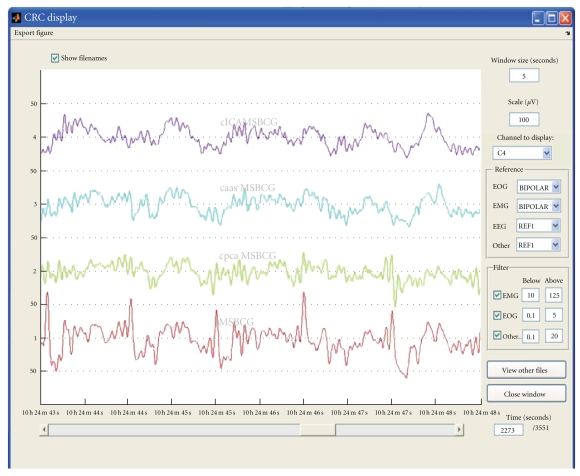
Comparison between the 3 correction methods, the signal displayed comes from electrode C4 and lasts 5 seconds: from top to bottom, EEG signal corrected by the cICA, AAS, and PCA method, and the original signal before correction.

One key step for the PA rejection is the detection of the heartbeats on one ECG channel. The method developed by Niazy and available in “the FMRIB plugin for EEGLAB” [8] is included in FA*𝕊*T. We found it very robust even on relatively noisy ECG channels. FA*𝕊*T currently provides five methods to reject the PA: “PCA” (from the FMRIB plugin), “Gaussian mean” (AAS from the FMRIB plugin) [12, 13], “constrained ICA (automatic)”, “constrained ICA (manual)” [7] and “AAS and PCA combined” (based on the FMRIB plugin). We would advise users with 30 channels or more to choose a “constrained ICA” (cICA) method. cICA was shown to be more efficient than AAS and PCA at rejecting the PA and to better preserve the spectrum of the “true” EEG signal [7]. This is particularly important when analyzing the time course of spontaneous activity (such as in sleep studies). See Figure 4 for an example of correction using AAS, PCA, and cICA on a stretch of EEG data acquired on a sleeping subject. It is difficult to pick the best correction just by looking at the corrected signal. 

With fewer channels (<30) or lots of movement activity, single-channel methods are better suited. “Gaussian mean” and PCA method do a good job in general. PCA is usually regarded as more efficient than “Gaussian mean” but Leclercq et al. [7] showed that for sleep EEG this is not the case: PCA tends to remove too much sleep activity, such as the slow waves which were picked up among the first few “optimal basis functions” and, therefore, removed for the recording. The “AAS and PCA combined” method is experimental and has not been rigorously tested. During the preparation of [7], we noticed that AAS was more efficient for the lower part of the data spectrum and PCA for the higher part. “AAS and PCA combined” thus uses a combination of AAS and PCA: AAS is applied to the low-pass filtered (≤4 Hz) signal and PCA on the high-pass filtered (≥4 Hz) signal, then the 2 corrected parts are recombined afterwards.

Users are in effect advised to test different correction methods to find out the most suitable one for their own data, depending on their final application and the usefulness of the validation criteria: here is a nonexhausitve list of proposed methods [11, 12, 14–20] which have been validated and applied on different types of data. Note that users are welcome to add other correction methods within FA*𝕊*T and hopefully share it with the other users.

### 3.3. Sleep Specific Tools

Specifically developed for the manual scoring of sleep M/EEG, these tools could be adapted to “scoring” other types of data. The aim is, therefore, to provide a user-friendly GUI to enter, for each time window of activity, a score via a set of predefined key presses. Scores provided by another mean, such as the automatic sleep scoring system ASEEGA [21, 22], can easily be added into the data structure, then displayed and used in FA*𝕊*T. Afterwards some summary statistics can also be calculated from the encoded score(s) and, in the case of sleep data, sleep slow waves can be automatically detected.

#### 3.3.1. Sleep Scoring and Statistics

**Figure 5:**
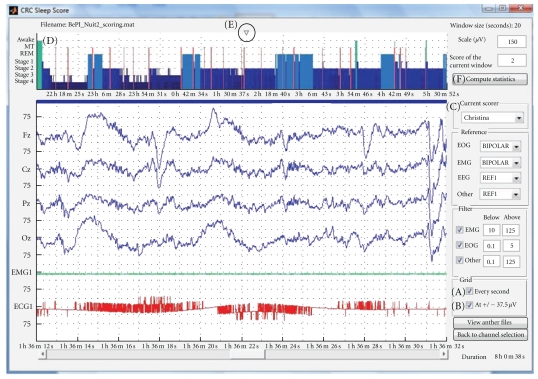
Sleep scoring GUI: (A) vertical grid toggle, (B) horizontal grid (±37 *μ*V toggle), (C) scorer selection, (D) hypnogram display, (E) current time position indicator, and (F) “compute sleep statistics” button.

This tool is similar to the simple visualization tool (Section 3.1.1) but lets the user manually attribute a “score” to any (fixed) time window of signal (see Figure 5). The same file may be scored by different users and their scorings reviewed later on.

The keypad is simply used to assign a score to the current window. Each number corresponds to a specific stage, by default: “wake state” (0), “sleep stage 1” (1), “sleep stage 2” (2), “sleep stage 3” (3), “sleep stage 4” (4), “REM sleep” (5), and “movement time” (6). Each time a score is assigned to the current window, the display moves on to the next time window. An hypnogram is automatically constructed along the scoring.

Other types of markers can also be added at any time: “artefact and arousal” (which will mark the window as artefacted for power spectrum computation) and “event of interest” (e.g., spindles, epileptic spikes, etc.). The “FPL marker”, that is, “closing door and light” marker (in French “fermer porte and lumière”), and the “OPL marker”, that is, “opening door and light” marker (in French “ouvrir porte and lumière”) specify the beginning and end of the “sleep recording” and are important to compute sleep statistics.

#### 3.3.2. Spectral Power Calculation and Display

**Figure 6:**
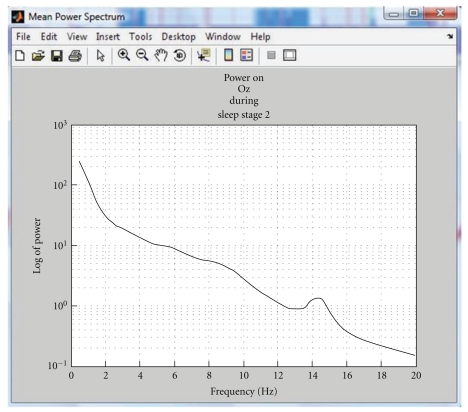
Mean power spectrum during a specific sleep stage (stage 2) for one channel (Oz here).

If the file was scored, then the spectral power is calculated as in Section 3.1.4, but sections scored as movement time or marked as artefacted are left out of the spectrogram calculation (power is set to zero). The hypnogram is also displayed (see Figure 3) alongside the spectrogram display. In the “frequency band” display mode, three scaling types are available: “absolute power”, “relative power”, that is, how much power is dissipated at time *t* in the considered band divided by the whole power at time *t*, and the “Mongrain view”, which shows the power dissipated at time *t* in the selected frequency band divided by the mean power in deep sleep stage during the night. Moreover, the mean power spectrum of one specific channel during a specific sleep stage can also be computed and displayed (see Figure 6).

#### 3.3.3. Slow Wave Detection

This tool, still in *BETA*-version, aims at automatically detecting slow waves (SWs) in sleep EEG recordings. It proceeds in successive steps: (1) extraction of the episode of interest, (2) bandpass filtering, (3) SW detection in four scalp “regions of interest” (ROIs), and (4) extraction of the SW trajectory on all electrodes.

**Figure 7:**
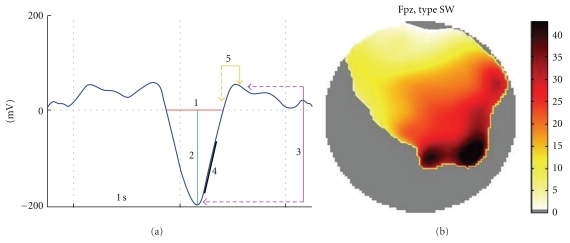
(a) SW detection criteria: (1) A negative zero crossing (downzero crossing) and a subsequent positive zero crossing (upzero crossing) separated by 0.25–1.25 sec, (2) A negative peak between the two zero crossings with voltage <−80 *μ*V, (3) A negative-to-positive peak-to-peak amplitude >140 *μ*V, (4) A positive slope >90% of the maximum slope, and (5) A positive zero crossing and a subsequent positive peak separated by maximum 2 sec. Right, display of a SW trajectory as map of delays.

The data episode to analyze can be the whole file, for example, if the data were previously chunked, or a part of the continuous file. This time window is either specified manually or relies on already defined sleep scores. To decrease the computational load, SWs detection is first performed on averaged signals from all the electrodes located in four scalp ROIs, by defaults: frontal, central left, central right, and parietal, that is, around the Fz, C3, C4, and Pz channels in the extended 10–20 system. SWs detection itself is performed in a spatiotemporal way following Massimini's criteria [23] which were adapted according to our observations on different data sets, see Figure 7(a).

The SW trajectory over the scalp is based on the temporal occurrence of the negative peak at all the electrodes, where the SW was detected. The SW “time delay” of each electrode, where a SW is detected, is defined as the difference between the negative peak time at this electrode and the negative peak time at the first electrode detecting the SW (Figure 7(b)). The characteristics of each SW are saved in the data structure for further use, and their occurrences are saved as “events” for an easy epoching of the data.

## 4. Conclusions and Perspectives

As stated earlier, we started writing this toolbox to process sleep EEG-fMRI data and tackle three crucial issues typical of this kind of data: data manipulation, fMRI-artefact rejection, and manual sleep-scoring. As far as we know, FA*𝕊*T is currently the only free toolbox that can deal with these specific issues in an efficient and flexible way on a standard computer. Nevertheless, FA*𝕊*T can certainly be a useful tool for other researchers in the EEG/MEG community, as data reviewing, marking, and handling are very common tasks.

Since FA*𝕊*T is the result of ongoing research project, more features and improvements are expected in the future: we are currently working on adding more sleep tools, such as an automatic spindle detection, and a better integration with SPM8 batching system. With the batch, the exact parameters used for one operation on a data set can be saved and reapplied, with or without modification, on any other data set. We are also open to suggestions and personal additions to the code. FA*𝕊*T is available here: http://www.montefiore.ulg.ac.be/~phillips/FASST.html/.
